# Dibromido(2,9-dimethyl-1,10-phenanthroline-κ^2^ *N*,*N*′)zinc

**DOI:** 10.1107/S1600536812022738

**Published:** 2012-05-26

**Authors:** Ali Dehghani, Mostafa M. Amini, Ezzatollah Najafi, Azadeh Tadjarodi, Behrouz Notash

**Affiliations:** a Department of Chemistry, Shahid Beheshti University, G.C., Evin, Tehran 1983963113, Iran; b Department of Chemistry, Iran University of Science and Technology, Tehran 16846-13114, Iran

## Abstract

The reaction of equimolar amounts of zinc bromide and 2,9-dimethyl-1,10-phenanthroline in dry methanol provided the title compound, [ZnBr_2_(C_14_H_12_N_2_)], in good yield. The Zn^II^ ion is coordinated in a distorted tetra­hedral environment by two N atoms from the chelating 2,9-dimethyl-1,10-phenanthroline ligand and two bromide ions. There is inter­molecular π–π stacking between adjacent phenanthroline units, with centroid–centroid distances of 3.594 (3) and 3.652 (3) Å.

## Related literature

For similiar structures, see: Seebacher *et al.* (2004 ▶); Harvey *et al.* (1999 ▶); Jordan *et al.* (1991 ▶); Pallenberg *et al.* (1997 ▶).
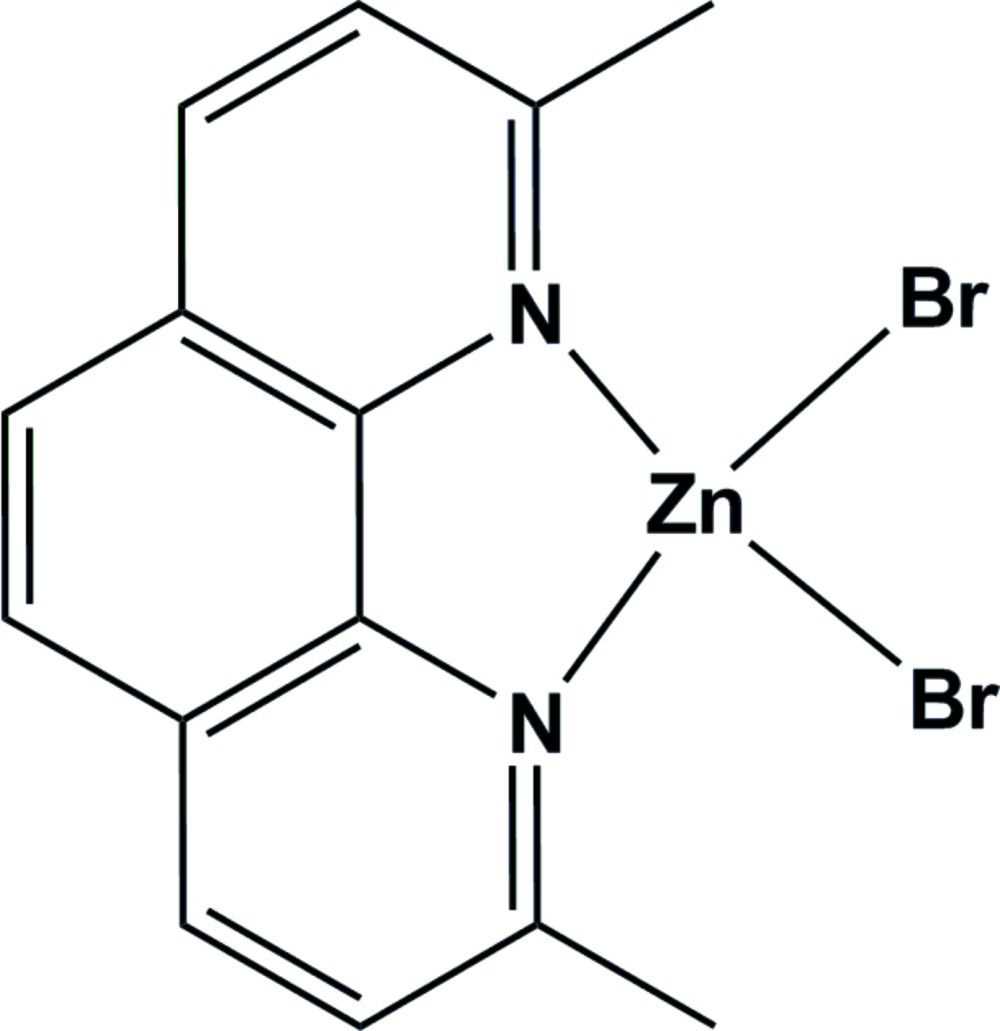


## Experimental

### 

#### Crystal data

[ZnBr_2_(C_14_H_12_N_2_)]
*M* *_r_* = 433.45
Monoclinic, 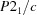

*a* = 9.4113 (19) Å
*b* = 18.424 (4) Å
*c* = 9.3362 (19) Å
β = 112.59 (3)°
*V* = 1494.6 (6) Å^3^
*Z* = 4
Mo *K*α radiation
μ = 6.98 mm^−1^
*T* = 298 K
0.25 × 0.20 × 0.17 mm

#### Data collection

Stoe IPDS 2T diffractometer
Absorption correction: numerical [shape of crystal determined optically (*X-RED32*; Stoe & Cie, (2005 ▶)] *T*_min_ = 0.274, *T*_max_ = 0.383
11850 measured reflections
4014 independent reflections
2304 reflections with *I* > 2σ(*I*)
*R*_int_ = 0.076

#### Refinement

*R*[*F* ^2^ > 2σ(*F* ^2^)] = 0.050
*wR*(*F* ^2^) = 0.100
*S* = 0.95
4014 reflections
174 parameters
H-atom parameters constrained
Δρ_max_ = 0.40 e Å^−3^
Δρ_min_ = −0.67 e Å^−3^

### 

Data collection: *X-AREA* (Stoe & Cie, 2005 ▶); cell refinement: *X-AREA*; data reduction: *X-AREA*; program(s) used to solve structure: *SHELXS97* (Sheldrick, 2008 ▶); program(s) used to refine structure: *SHELXL97* (Sheldrick, 2008 ▶); molecular graphics: *ORTEP-3 for Windows* (Farrugia, 1997 ▶); software used to prepare material for publication: *WinGX* (Farrugia, 1999 ▶).

## Supplementary Material

Crystal structure: contains datablock(s) I, global. DOI: 10.1107/S1600536812022738/bt5921sup1.cif

Structure factors: contains datablock(s) I. DOI: 10.1107/S1600536812022738/bt5921Isup2.hkl

Additional supplementary materials:  crystallographic information; 3D view; checkCIF report

## Figures and Tables

**Fig. 1.**
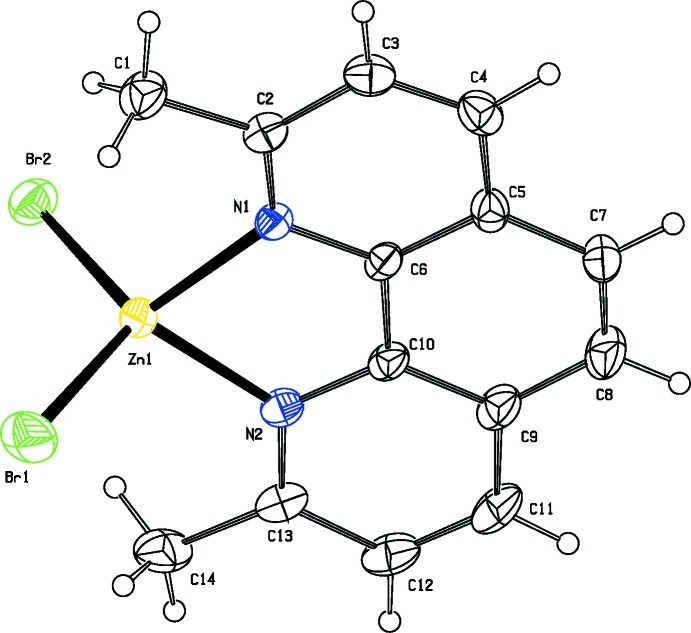
The molecular structure of the title compound, ellipsoids drawn at 30% probability level.

**Fig. 2.**
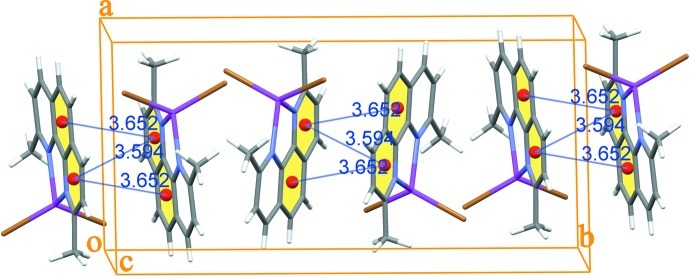
The packing diagram of the title compound showing π–π stacking between adjacent 2,9-dimethyl-1,10-phenanthroline ligands.

## supplementary crystallographic information

### Comment

1,10-phenanthroline is a good bidentate chelating ligand, we present the crystal structure of the title complex based on 2,9-dimethyl-1,10-phenanthroline.

In the molecule of the title compound, (Fig. 1), the two N atoms of one phen ligand and two Br atoms are coordinated to Zn^II^ atom in a distorted tetrahedral arrangement. The Zn—N bonds [average 2.062 Å] are somewhat shorter than the Zn—Br distances [average 2.328 Å] and they are closed to such bond lengths found in other discrete 1,10-phenanthroline derivatives of zinc complexes (Seebacher *et al.*, (2004); Harvey *et al.*,(1999)). The two N atoms bite angle of phen ligand, N(2)—Zn(1)—N(1), significantly is smaller than N(2)—Zn(1)—Br(1)and N(1)—Zn(1)—Br(2). The bite angle in title complex is also similar to that of found in other zinc complexes of 1,10-phenanthroline, regardless of geometry of complex (Jordan *et al.*,(1991); Pallenberg *et al.*,(1997)).

In the crystal structure, There are intermolecular π–π stacking between adjacent phenanthroline, with a centroid–centroid distances of 3.594 (3) and 3.652 (3) Å (Fig. 2). These π-π stacking interactions lead to the stabilization of the crystal structure.

### Experimental

ZnBr_2_.2H_2_O (0.22 g, 1 mmol) and 2,9-dimethyl-1,10-phenanthroline (0.21, 1 mmol) were loaded in a convection tube; the tube was filled with methanol and kept at 333 K. Colorless crystals were collected from the side arm after several days(m.p. > 543 K).

### Refinement

The C—H protons were positioned geometrically and refined as riding atoms with C—H = 0.93 Å and *U*iso(H) = 1.2 *U*eq(C) for aromatic C—H groups, C—H = 0.96 Å and *U*iso(H) = 1.5 *U*eq(C) for methyl groups.

### Crystal data

**Table:**

[ZnBr_2_(C_14_H_12_N_2_)]	*F*(000) = 840
*M**_r_* = 433.45	*D*_x_ = 1.926 Mg m^−^^3^
Monoclinic, *P*2_1_/*c*	Mo *K*α radiation, λ = 0.71073 Å
Hall symbol: -P 2ybc	Cell parameters from 4014 reflections
*a* = 9.4113 (19) Å	θ = 2.2–29.2°
*b* = 18.424 (4) Å	µ = 6.98 mm^−^^1^
*c* = 9.3362 (19) Å	*T* = 298 K
β = 112.59 (3)°	Block, colorless
*V* = 1494.6 (6) Å^3^	0.25 × 0.20 × 0.17 mm
*Z* = 4	

### Data collection

**Table:**

Stoe IPDS 2T diffractometer	4014 independent reflections
Radiation source: fine-focus sealed tube	2304 reflections with *I* > 2σ(*I*)
Graphite monochromator	*R*_int_ = 0.076
Detector resolution: 0.15 mm pixels mm^-1^	θ_max_ = 29.2°, θ_min_ = 2.2°
rotation method scans	*h* = −12→12
Absorption correction: numerical [shape of crystal determined optically (*X-RED32*; Stoe & Cie, (2005)]	*k* = −23→25
*T*_min_ = 0.274, *T*_max_ = 0.383	*l* = −12→12
11850 measured reflections	

### Refinement

**Table:**

Refinement on *F*^2^	Primary atom site location: structure-invariant direct methods
Least-squares matrix: full	Secondary atom site location: difference Fourier map
*R*[*F*^2^ > 2σ(*F*^2^)] = 0.050	Hydrogen site location: inferred from neighbouring sites
*wR*(*F*^2^) = 0.100	H-atom parameters constrained
*S* = 0.95	*w* = 1/[σ^2^(*F*_o_^2^) + (0.0423*P*)^2^] where *P* = (*F*_o_^2^ + 2*F*_c_^2^)/3
4014 reflections	(Δ/σ)_max_ < 0.001
174 parameters	Δρ_max_ = 0.40 e Å^−^^3^
0 restraints	Δρ_min_ = −0.67 e Å^−^^3^

### Special details

**Table:**

Geometry. All e.s.d.'s (except the e.s.d. in the dihedral angle between two l.s. planes) are estimated using the full covariance matrix. The cell e.s.d.'s are taken into account individually in the estimation of e.s.d.'s in distances, angles and torsion angles; correlations between e.s.d.'s in cell parameters are only used when they are defined by crystal symmetry. An approximate (isotropic) treatment of cell e.s.d.'s is used for estimating e.s.d.'s involving l.s. planes.
Refinement. Refinement of *F*^2^ against ALL reflections. The weighted *R*-factor *wR* and goodness of fit *S* are based on *F*^2^, conventional *R*-factors *R* are based on *F*, with *F* set to zero for negative *F*^2^. The threshold expression of *F*^2^ > σ(*F*^2^) is used only for calculating *R*-factors(gt) *etc*. and is not relevant to the choice of reflections for refinement. *R*-factors based on *F*^2^ are statistically about twice as large as those based on *F*, and *R*- factors based on ALL data will be even larger.

### Fractional atomic coordinates and isotropic or equivalent isotropic displacement parameters (Å^2^)

**Table:**

	*x*	*y*	*z*	*U*_iso_*/*U*_eq_	
Zn1	0.66996 (6)	0.13233 (3)	0.75289 (6)	0.03618 (15)	
Br1	0.79790 (8)	0.24221 (4)	0.77098 (8)	0.0667 (2)	
Br2	0.74325 (7)	0.03299 (3)	0.63755 (6)	0.05122 (17)	
N1	0.6409 (4)	0.1030 (2)	0.9541 (4)	0.0317 (8)	
N2	0.4348 (4)	0.1459 (2)	0.6749 (4)	0.0349 (9)	
C1	0.9118 (6)	0.0852 (3)	1.1082 (6)	0.0514 (14)	
H1A	0.9226	0.0590	1.0240	0.077*	
H1B	0.9740	0.0627	1.2047	0.077*	
H1C	0.9447	0.1345	1.1074	0.077*	
C2	0.7466 (5)	0.0842 (3)	1.0900 (5)	0.0359 (10)	
C3	0.7030 (6)	0.0634 (3)	1.2131 (5)	0.0429 (12)	
H3	0.7775	0.0492	1.3077	0.051*	
C4	0.5507 (6)	0.0641 (3)	1.1932 (6)	0.0434 (12)	
H4	0.5217	0.0492	1.2733	0.052*	
C5	0.4398 (6)	0.0872 (3)	1.0528 (5)	0.0376 (11)	
C6	0.4904 (5)	0.1057 (2)	0.9352 (5)	0.0318 (10)	
C7	0.2784 (7)	0.0920 (3)	1.0226 (7)	0.0479 (13)	
H7	0.2437	0.0787	1.0996	0.058*	
C8	0.1774 (6)	0.1151 (3)	0.8858 (7)	0.0561 (15)	
H8	0.0740	0.1188	0.8706	0.067*	
C9	0.2244 (6)	0.1343 (3)	0.7625 (6)	0.0442 (12)	
C10	0.3796 (5)	0.1290 (3)	0.7864 (5)	0.0346 (10)	
C11	0.1239 (6)	0.1607 (3)	0.6167 (7)	0.0557 (14)	
H11	0.0195	0.1660	0.5956	0.067*	
C12	0.1803 (6)	0.1781 (3)	0.5082 (6)	0.0549 (14)	
H12	0.1140	0.1956	0.4124	0.066*	
C13	0.3386 (6)	0.1702 (3)	0.5378 (6)	0.0442 (12)	
C14	0.4045 (7)	0.1888 (3)	0.4201 (6)	0.0588 (15)	
H14A	0.4686	0.2310	0.4535	0.088*	
H14B	0.3224	0.1985	0.3222	0.088*	
H14C	0.4648	0.1488	0.4089	0.088*	

### Atomic displacement parameters (Å^2^)

**Table:**

	*U*^11^	*U*^22^	*U*^33^	*U*^12^	*U*^13^	*U*^23^
Zn1	0.0290 (3)	0.0397 (3)	0.0402 (3)	0.0015 (3)	0.0137 (2)	0.0058 (2)
Br1	0.0542 (4)	0.0502 (4)	0.0879 (5)	−0.0154 (3)	0.0186 (3)	0.0098 (3)
Br2	0.0539 (4)	0.0554 (4)	0.0464 (3)	0.0142 (3)	0.0216 (3)	0.0027 (3)
N1	0.028 (2)	0.032 (2)	0.035 (2)	0.0023 (17)	0.0115 (17)	0.0016 (16)
N2	0.032 (2)	0.035 (2)	0.0329 (19)	0.0022 (18)	0.0073 (16)	−0.0006 (17)
C1	0.037 (3)	0.062 (4)	0.050 (3)	0.005 (3)	0.011 (3)	0.003 (3)
C2	0.033 (3)	0.034 (3)	0.035 (2)	0.001 (2)	0.007 (2)	−0.0042 (19)
C3	0.049 (3)	0.041 (3)	0.033 (2)	0.002 (3)	0.008 (2)	0.000 (2)
C4	0.056 (3)	0.041 (3)	0.039 (3)	−0.002 (3)	0.024 (3)	0.000 (2)
C5	0.039 (3)	0.032 (3)	0.047 (3)	−0.005 (2)	0.023 (2)	−0.005 (2)
C6	0.025 (2)	0.027 (2)	0.042 (2)	0.0005 (19)	0.012 (2)	−0.0028 (19)
C7	0.046 (3)	0.046 (3)	0.064 (3)	0.000 (3)	0.034 (3)	0.004 (3)
C8	0.030 (3)	0.061 (4)	0.080 (4)	−0.004 (3)	0.024 (3)	0.002 (3)
C9	0.028 (2)	0.045 (3)	0.056 (3)	0.001 (2)	0.011 (2)	−0.002 (3)
C10	0.024 (2)	0.031 (3)	0.044 (2)	0.000 (2)	0.0077 (19)	−0.001 (2)
C11	0.025 (3)	0.065 (4)	0.066 (4)	0.011 (3)	0.004 (2)	−0.002 (3)
C12	0.039 (3)	0.058 (4)	0.047 (3)	0.012 (3)	−0.006 (2)	0.007 (3)
C13	0.043 (3)	0.037 (3)	0.044 (3)	0.010 (2)	0.007 (2)	−0.001 (2)
C14	0.066 (4)	0.061 (4)	0.039 (3)	0.013 (3)	0.010 (3)	0.013 (3)

### Geometric parameters (Å, º)

**Table:**

Zn1—N2	2.062 (4)	C5—C6	1.396 (6)
Zn1—N1	2.071 (3)	C5—C7	1.437 (7)
Zn1—Br1	2.3281 (9)	C6—C10	1.445 (7)
Zn1—Br2	2.3572 (8)	C7—C8	1.336 (8)
N1—C2	1.322 (6)	C7—H7	0.9300
N1—C6	1.360 (6)	C8—C9	1.427 (7)
N2—C13	1.329 (6)	C8—H8	0.9300
N2—C10	1.366 (5)	C9—C10	1.394 (7)
C1—C2	1.498 (7)	C9—C11	1.412 (8)
C1—H1A	0.9600	C11—C12	1.351 (7)
C1—H1B	0.9600	C11—H11	0.9300
C1—H1C	0.9600	C12—C13	1.414 (7)
C2—C3	1.414 (6)	C12—H12	0.9300
C3—C4	1.372 (7)	C13—C14	1.494 (7)
C3—H3	0.9300	C14—H14A	0.9600
C4—C5	1.392 (7)	C14—H14B	0.9600
C4—H4	0.9300	C14—H14C	0.9600
			
N2—Zn1—N1	81.63 (14)	N1—C6—C5	122.8 (4)
N2—Zn1—Br1	112.03 (11)	N1—C6—C10	117.8 (4)
N1—Zn1—Br1	113.94 (11)	C5—C6—C10	119.4 (4)
N2—Zn1—Br2	113.29 (11)	C8—C7—C5	121.2 (4)
N1—Zn1—Br2	112.05 (11)	C8—C7—H7	119.4
Br1—Zn1—Br2	118.32 (3)	C5—C7—H7	119.4
C2—N1—C6	119.7 (4)	C7—C8—C9	121.5 (5)
C2—N1—Zn1	128.7 (3)	C7—C8—H8	119.2
C6—N1—Zn1	111.5 (3)	C9—C8—H8	119.2
C13—N2—C10	119.5 (4)	C10—C9—C11	116.8 (4)
C13—N2—Zn1	128.5 (3)	C10—C9—C8	118.9 (5)
C10—N2—Zn1	112.0 (3)	C11—C9—C8	124.2 (5)
C2—C1—H1A	109.5	N2—C10—C9	123.0 (4)
C2—C1—H1B	109.5	N2—C10—C6	117.1 (4)
H1A—C1—H1B	109.5	C9—C10—C6	119.9 (4)
C2—C1—H1C	109.5	C12—C11—C9	119.6 (5)
H1A—C1—H1C	109.5	C12—C11—H11	120.2
H1B—C1—H1C	109.5	C9—C11—H11	120.2
N1—C2—C3	120.3 (4)	C11—C12—C13	121.1 (5)
N1—C2—C1	118.1 (4)	C11—C12—H12	119.5
C3—C2—C1	121.6 (4)	C13—C12—H12	119.5
C4—C3—C2	120.1 (5)	N2—C13—C12	120.0 (5)
C4—C3—H3	120.0	N2—C13—C14	117.6 (5)
C2—C3—H3	120.0	C12—C13—C14	122.4 (5)
C3—C4—C5	119.8 (4)	C13—C14—H14A	109.5
C3—C4—H4	120.1	C13—C14—H14B	109.5
C5—C4—H4	120.1	H14A—C14—H14B	109.5
C4—C5—C6	117.1 (4)	C13—C14—H14C	109.5
C4—C5—C7	123.9 (4)	H14A—C14—H14C	109.5
C6—C5—C7	119.0 (5)	H14B—C14—H14C	109.5
			
N2—Zn1—N1—C2	−177.9 (4)	C7—C5—C6—C10	−0.1 (7)
Br1—Zn1—N1—C2	−67.4 (4)	C4—C5—C7—C8	179.4 (5)
Br2—Zn1—N1—C2	70.3 (4)	C6—C5—C7—C8	−1.5 (8)
N2—Zn1—N1—C6	1.1 (3)	C5—C7—C8—C9	1.7 (9)
Br1—Zn1—N1—C6	111.6 (3)	C7—C8—C9—C10	−0.3 (9)
Br2—Zn1—N1—C6	−110.7 (3)	C7—C8—C9—C11	−178.5 (6)
N1—Zn1—N2—C13	176.8 (4)	C13—N2—C10—C9	1.5 (7)
Br1—Zn1—N2—C13	64.3 (4)	Zn1—N2—C10—C9	179.4 (4)
Br2—Zn1—N2—C13	−72.7 (4)	C13—N2—C10—C6	−177.5 (4)
N1—Zn1—N2—C10	−0.8 (3)	Zn1—N2—C10—C6	0.4 (5)
Br1—Zn1—N2—C10	−113.3 (3)	C11—C9—C10—N2	−1.9 (8)
Br2—Zn1—N2—C10	109.7 (3)	C8—C9—C10—N2	179.8 (5)
C6—N1—C2—C3	3.3 (7)	C11—C9—C10—C6	177.1 (5)
Zn1—N1—C2—C3	−177.8 (3)	C8—C9—C10—C6	−1.3 (8)
C6—N1—C2—C1	−177.3 (4)	N1—C6—C10—N2	0.6 (6)
Zn1—N1—C2—C1	1.7 (7)	C5—C6—C10—N2	−179.5 (4)
N1—C2—C3—C4	−1.5 (7)	N1—C6—C10—C9	−178.4 (4)
C1—C2—C3—C4	179.1 (5)	C5—C6—C10—C9	1.4 (7)
C2—C3—C4—C5	−1.7 (8)	C10—C9—C11—C12	1.0 (8)
C3—C4—C5—C6	2.8 (7)	C8—C9—C11—C12	179.2 (6)
C3—C4—C5—C7	−178.0 (5)	C9—C11—C12—C13	0.2 (9)
C2—N1—C6—C5	−2.0 (7)	C10—N2—C13—C12	−0.2 (7)
Zn1—N1—C6—C5	178.9 (4)	Zn1—N2—C13—C12	−177.7 (4)
C2—N1—C6—C10	177.8 (4)	C10—N2—C13—C14	179.4 (4)
Zn1—N1—C6—C10	−1.3 (5)	Zn1—N2—C13—C14	1.9 (7)
C4—C5—C6—N1	−1.1 (7)	C11—C12—C13—N2	−0.7 (9)
C7—C5—C6—N1	179.8 (5)	C11—C12—C13—C14	179.8 (6)
C4—C5—C6—C10	179.1 (4)		

## References

[bb1] Farrugia, L. J. (1997). *J. Appl. Cryst.* **30**, 565.

[bb2] Farrugia, L. J. (1999). *J. Appl. Cryst.* **32**, 837–838.

[bb3] Harvey, M., Baggio, S., Baggio, R. & Mombrú, A. W. (1999). *Acta Cryst.* C**55**, 308–310.

[bb4] Jordan, K. J., Wacholtz, W. F. & Crosby, G. A. (1991). *Inorg. Chem.* **30**, 4588–4593.

[bb5] Pallenberg, A. J., Marschner, T. M. & Barnhart, D. M. (1997). *Polyhedron*, **16**, 2711–2719.

[bb6] Seebacher, J., Mian, J. & Vahrenkamp, H. (2004). *Eur. J. Inorg. Chem.* pp. 409–417.

[bb7] Sheldrick, G. M. (2008). *Acta Cryst.* A**64**, 112–122. 10.1107/S0108767307043930 18156677

[bb8] Stoe & Cie (2005). *X-AREA* and *X-RED32* Stoe & Cie, Darmstadt, Germany.

